# Recent advances in understanding and managing self-harm in adolescents

**DOI:** 10.12688/f1000research.19868.1

**Published:** 2019-10-24

**Authors:** Stephanie Clarke, Lauren A. Allerhand, Michele S. Berk

**Affiliations:** 1 Department of Psychiatry & Behavioral Sciences, Stanford University School of Medicine, 401 Quarry Road, Stanford, CA, 94305-5719, USA

**Keywords:** suicide, adolescent, self-harm

## Abstract

Adolescent suicide is a serious public health problem, and non-suicidal self-injury (NSSI) is both highly comorbid with suicidality among adolescents and a significant predictor of suicide attempts (SAs) in adolescents. We will clarify extant definitions related to suicidality and NSSI and the important similarities and differences between these constructs. We will also review several significant risk factors for suicidality, evidence-based and evidence-informed safety management strategies, and evidence-based treatment for adolescent self-harming behaviors. Currently, dialectical behavior therapy (DBT) for adolescents is the first and only treatment meeting the threshold of a well-established treatment for self-harming adolescents at high risk for suicide. Areas in need of future study include processes underlying the association between NSSI and SAs, clarification of warning signs and risk factors that are both sensitive and specific enough to accurately predict who is at imminent risk for suicide, and further efforts to sustain the effects of DBT post-treatment. DBT is a time- and labor-intensive treatment that requires extensive training for therapists and a significant time commitment for families (generally 6 months). It will therefore be helpful to assess whether other less-intensive treatment options can be established as evidence-based treatment for suicidal adolescents.

## Introduction

Suicide is a significant public health problem in the United States. Over the last 15 years, suicide rates have increased across all age groups; however, the greatest increase has been found in females aged 10–14 (200%) ^1, 2^. Suicide is the second leading cause of death among 10–24 year olds ^3^. Despite decades of research on suicide prevention in this age group, suicide rates among 10–19 year olds increased by 56% between 2007 and 2016. A 2017 national survey found that 17.2% of high school students seriously considered making a suicide attempt (SA), 13.6% made a suicide plan, and 7.4% made one or more SAs ^4^. Historically, males are more likely to die by suicide; however, incident rate ratios comparing deaths by suicide between adolescent males and females have been decreasing since 2007, with findings demonstrating that numbers of adolescent female suicides are catching up to those of their male counterparts ^2^. An alarmingly large percentage of adolescents experience suicidal ideation (SI), and SI has been shown to confer significant risk for future SAs. Adolescents who experience SI are 12 times more likely than adolescents who do not experience SI to attempt suicide by age 30, with 86.1% of adolescents making a SA within 12 months of SI onset ^5^. More than one-third of adolescents who experience SI go on to attempt suicide ^5^.

Rates of non-suicidal self-injury (NSSI; e.g. scratching or cutting oneself; see below) are also high, particularly among adolescents, with approximately 18% of high school students overall and 24% of high school girls in the U.S. reporting at least one episode of NSSI within the past 12 months ^6^. While, by definition, the purpose of NSSI is not to end one’s life (see below), NSSI is a significant risk factor for future SAs ^7, 8^. We will describe important differences and similarities among types of self-injurious behaviors (i.e. NSSI and SA), risk factors for self-injury among adolescents, and evidence-based management and treatment intervention strategies for these behaviors.

## Definitions of self-injurious behaviors

SAs and NSSI can be grouped into the overarching category of self-harm (SH). SAs are potentially self-injurious behaviors conducted *with some intent to die* (i.e. to cause one’s own death) as a result of the behavior ^9^. NSSIs are self-injurious behaviors performed *without any intent to die* ^9^ and include such behaviors as scratching the skin, cutting, burning, head banging, hitting oneself, and so forth ^9^. The purposes, or functions, of NSSI are typically to reduce or distract from negative emotions, punish oneself, and/or reduce feelings of numbness or dissociation ^10^. While some studies have examined SA and NSSI as separate outcomes, differentiated by the presence (SAs) or absence (NSSI) of intent to die as a result of the behavior, other studies have used the broader category of SH, in part, because of the challenge of accurately determining whether or not SH included an intent to die ^11^. Inconsistencies in how SH and suicidal behaviors are studied—as an overall category of SH versus separate entities of NSSI and SA—has made it difficult to compare outcomes between studies and has therefore hampered progress in furthering our understanding of these constructs. See Table 1 for a list of terms, abbreviations, and definitions of SH used in this review.

**Table 1.** Definitions of types of self-injurious behaviors.

Term	Abbreviation	Definition
Suicide attempt	SA	Potentially self-injurious behaviors conducted deliberately *with some intent to die* (i.e. to cause one’s own death) as a result of the behavior ^8^.
Non-suicidal self-injury	NSSI	Self-injurious behaviors performed deliberately *without any intent to die* ^8^. Common methods of NSSI include scratching the skin, cutting, burning, head banging, and hitting oneself ^9^.
Self-harm	SH	Broader category including all intentional self-injury, with or without intent to die (i.e. SA and NSSI).

## Similarities and differences between suicide attempt and non-suicidal self-injury

Research in recent years has focused on the similarities and differences between SAs and NSSI. As noted above, SA and NSSI are similar in that both are types of deliberate SH. While the major differentiator between NSSI and SAs is the presence or absence of intent to die, other factors differentiate these behaviors (e.g. function, lethality, medical severity, prevalence rate, and frequency of the behavior; see Table 1) ^12, 13^. SAs and NSSI were previously addressed in the *Diagnostic and Statistical Manual of Mental Disorders* (DSM) IV only as symptoms of other mental disorders, such as major depressive disorder (MDD) and borderline personality disorder (BPD) ^14^. DSM 5 ^14^ includes new diagnostic categories, NSSI disorder and suicidal behavior disorder, as “condition[s] for further study” ^14^. The inclusion of these disorders increases consistency with the extant treatment literature, which generally holds that NSSI and SAs must be targeted directly, rather than as secondary to other diagnoses. This is in opposition to views that NSSI and suicidality are symptoms of other disorders, such as depressive disorders, that will remit independently as depression is treated ^15^.

While there are important distinctions between SAs and NSSI, the relationship between these behaviors is important. Research shows that most adolescents engage in NSSI and SAs concurrently ^16^. In fact, the majority of adolescents who have engaged in NSSI have also attempted suicide ^17^, and NSSI is a robust predictor of future SA among youth with depressive disorders ^7, 8^. Recent findings elucidated a temporal order to SI, SAs, and NSSI, such that adolescents have already thought about suicide prior to the first onset of NSSI, and NSSI typically precedes the first SA ^13^. It has been posited that NSSI serves as a “gateway” to SA by way of eroding natural tendencies of self-preservation and increasing comfort with SH ^16^.

## Risk factors for suicidal behavior in adolescents

In November of 2016, the American Psychological Association (APA) released a statement titled, “After Decades of Research, Science Is No Better Able to Predict Suicidal Behaviors” ^18^. This was based on a meta-analysis of 50 years of research on risk factors for suicidality in which the authors found that the ability of identified risk factors to predict suicidal thoughts and behaviors was only slightly better than chance ^19^. At this time, there is not a standardized suicide risk assessment that accurately predicts suicidal behavior. Currently, any attempt to apply a standardized risk assessment to determine who is at imminent risk for suicide will yield a high number of false positives ^20^. This is because many of the risk factors for suicide are also found in individuals who are not suicidal; in other words, identified risk factors and warning signs for suicide are neither sensitive nor specific enough to accurately predict who is at imminent risk for suicide ^21, 22^. While it has generally been determined that risk for suicidal behavior increases with the number of risk factors present ^23^, it is also not yet known what combinations of risk factors are most likely to lead to suicidal behavior and death by suicide ^19^. While a full review of risk factors for suicidality are beyond the scope of this paper, the adolescent literature has identified several risk factors for suicidal behavior, many of which—but not all—are listed in Table 2.

**Table 2.** Risk factors for suicidal behavior and deaths by suicide.

Risk factor
Suicidal ideation ^23, 47– 49^
Previous suicide attempt ^23, 50– 52^
Suicide intent (i.e. extent to which an individual wishes to die) ^51, 52^
Non-suicidal self-injury ^7, 46, 50, 53, 54^
Precipitating events
e.g. family conflict ^25, 55– 57^; loss of parent through death or divorce or living away from one or both parents ^56, 58, 59^; other interpersonal conflict ^60^; being a victim or perpetrator of bullying ^61– 64^
Sexual orientation and gender identity ^65– 70^
Psychopathology
e.g. MDD ^71, 72^/MDD severity ^73– 75^/symptoms of MDD, such as feelings of worthlessness ^73^, hopelessness ^76, 77^, and low positive expectancies ^76^; bipolar disorders ^78^; alcohol use disorder and other substance use disorders ^49^; anxiety disorders ^52, 79^; post- traumatic stress disorder ^52, 79^; psychosis ^52, 79^; eating disorders ^52, 79^; ADHD ^52, 78– 80^; conduct disorder ^52, 80, 81^; personality disorders and characteristics (e.g. antisocial, borderline, histrionic, and narcissistic personality disorders); psychiatric comorbidity (i.e. more than one psychiatric disorder) ^56, 82^
Psychological and personality factors
e.g. impulsivity ^83– 85^; impulsive aggression ^86, 87^; neuroticism ^87– 90^; perceived burdensomeness ^91^; poor coping and problem-solving abilities ^24^; low self-esteem ^58^; high levels of anger ^23, 86^; perception of expectation of perfectionism ^92^; negative self-referential thinking (i.e. processing bias that over-emphasizes negative information) and negative inferential style (i.e. believing negative events predict negative outcomes in the future) ^93^
Sleep problems ^93– 95^
Family history of suicide ^96– 99^
Childhood maltreatment ^100– 105^
Psychiatric hospitalization ^37, 106^
Contagion ^107– 112^

ADHD, attention-deficit hyperactivity disorder; MDD, major depressive disorder

In recent years, research has aimed to elucidate which risk factors are modifiable ^24^ (i.e. can be changed) and when they occur in time prior to suicidal behavior ^19^. With this information, clinicians will be able to target dynamic risk factors (e.g. family conflict) ^25^ for suicidal behavior more accurately in order to reduce suicide risk. Furthermore, identifying proximal risk factors (e.g. sleep disturbance) ^26^ will help clinicians determine who is at immediate risk of engaging in suicidal behavior. Presently, there are several websites with lists of warning signs (i.e. behavioral indicators that a SA is imminent or likely to happen within hours to days) ^27^ for adolescent suicidal behavior; however, there is little research to support these as indicators of imminent suicidal behavior.

## Strategies for managing suicidal youth in outpatient settings

There are several strategies that can be used across diagnoses and outpatient treatments to manage safety concerns when working with suicidal adolescents and their families.

### Written safety plan

Because patients may have difficulty identifying and implementing adaptive coping strategies in the place of SH when they are in the midst of a suicidal crisis and overwhelmed by strong negative emotions, the written safety plan helps the suicidal individual to select and use coping strategies in crisis situations ^28– 31^. Safety plans have been used in the context of short-term, empirically supported treatments that have been found to reduce suicide risk, such as cognitive therapy ^31, 32^ and cognitive behavior therapy for suicide prevention (CBT-SP) ^30^. It is critical to note that the written safety plan is not a “no-suicide” contract, which has been shown to be ineffective in preventing suicidal behavior and death by suicide ^33, 34^.

### Means restriction

Means restriction includes removing or limiting access to potentially life-threatening settings or objects, such as firearms, tall buildings, bridges, trains/traffic, sharps, and any medications and toxic substances that could be used to overdose ^35^. It has been documented that removing the suicidal individual’s access to lethal means is a highly effective suicide prevention strategy with a robust empirical basis ^36, 37^. Access to firearms, in particular, is a significant predictor of death by suicide, independent of all other factors ^38^. Of note, between 1996 and 2010, firearms were the most common method used in death by suicide in the U.S. (51%) ^39^. This statistic is underscored by findings demonstrating that limited access to firearms explains the significantly smaller number of deaths by suicide among on-campus college students, who have a nearly ninefold decrease in access to firearms when compared to age- and gender-matched controls ^40^. Furthermore, psychological autopsy studies of adolescents who died by suicide in the absence of psychopathology have suggested that access to a loaded firearm is a significant risk factor for death by suicide ^41^. In states within the U.S. and other countries, greater firearm regulations are associated with reductions in suicide deaths by firearms ^42^.

### Increased monitoring/supervision

Several studies have linked low parental supervision and monitoring (i.e. physical supervision of the adolescent as well as awareness of the adolescent’s activities and schedule) to increased risk for suicidal behavior among adolescents ^43, 44^. The American Academy of Child and Adolescent Psychiatry (AACAP) practice parameter for suicidal youth recommend that suicidal children and adolescents be monitored closely by a trustworthy and supportive adult ^45^.

### Reducing risk factors

Reducing malleable risk factors related to adolescent suicide, such as decreasing family conflict, seeking treatment for sleep problems, and addressing peer victimization and bullying with the school, requires parental involvement.

### Working with parents

Strengthening the parent–teen relationship and improving family functioning are critical when treating suicidal youth ^31^. We ultimately want the adolescent to communicate with parents when they are in a suicidal crisis so that parents can help keep the adolescent safe and obtain appropriate help. Additionally, family conflict is a known risk factor for adolescent suicide and suicidal behavior while family cohesion serves as a protective factor against suicide and suicidal behavior ^46^. Finally, parents have a significant role in implementing strategies outside of treatment to try to prevent the adolescent from making a SA, such as removing access to lethal means, providing supervision and monitoring, and seeking emergency services when needed.

### Treatment approaches

A 2015 meta-analysis of 19 studies of therapeutic interventions (TIs) for SH behavior in adolescents found TIs to be superior to control conditions for decreasing SH. The strongest effect sizes were found for DBT, mentalization-based therapy (MBT), and CBT ^113^. As noted earlier, working with parents as part of the treatment of teens who SH is of critical importance. The same meta-analysis also found that effect sizes were greatest for treatments that included family-based components ^53^. As previously mentioned, family conflict is a significant risk factor for suicidal behavior in adolescents ^25, 55– 57^ and must be addressed when working with suicidal youth. Additionally, the relationship between the suicidal adolescent and parent or caregiver is important given that, as discussed above, parents play a crucial role in safety planning.

A 2018 systematic review examined 21 randomized controlled trials (RCTs) and assessed 18 distinct treatment interventions, five of which reported significant results in reducing SH or SAs when compared to treatment as usual ^114^. These five interventions included DBT for adolescents, MBT, safe alternatives for teens and youth, integrated CBT, and developmental group psychotherapy (DGP). Two replication studies of DGP did not find significant results. Common elements of the five interventions with significant results included family involvement or support, emotion regulation skills, communication skills, and problem-solving skills ^114^.

A 2019 review of 26 RCTs published prior to June 2018 found nine new studies since the 2015 review discussed above ^115^. Integrated family therapy also joined other interventions with RCTs yielding significant results. The most significant change was that DBT, based on a second RCT with significant results from a different research group, meets the threshold of a “well-established” ^116^ treatment for reducing SH.

### Dialectical behavior therapy

At this time, DBT is the first and only “well-established” treatment for suicidal and SH adolescents. DBT targets both SA and NSSI by identifying the function of the behavior (e.g. reducing emotional distress) for the given individual and finding ways to obtain that function safely using DBT-based coping skills. Components of standard DBT are the same for adolescents and adults (see Table 3), with the exception of there being parenting and family sessions with the individual therapist as needed and the skills class including both teens and parents ^117– 119^.

**Table 3.** Components of stage I standard dialectical behavior therapy for adolescents ^116– 118^.

Component	Function	Structure
Individual psychotherapy (At least 1 per week)	1) Enhance capacities related to skills modules 2) Skills application to patient’s unique circumstances 3) Improve motivation and reduce dysfunctional behavior 4) Structure the environment to reinforce effective behavior and positive change	Treatment hierarchy: 1) Life-threatening behavior 2) Therapy-interfering behavior 3) Quality-of-life interfering behavior
Multifamily group skills training (1 per week)	Teach skills: 1) Mindfulness 2) Distress tolerance 3) Emotion regulation 4) Interpersonal effectiveness 5) Middle path skills	1) Mindfulness exercise 2) Homework review 3) Teaching of new skill
Telephone coaching (Available 24/7 for youth and parents)	1) Help with skills application in context (e.g. in a crisis) 2) Unavailable for 24 hours after patient engages in self- injurious behavior	Brief, focused calls for 1) Skill use in a crisis 2) Addressing therapist–patient rupture 3) Reporting good news
Therapist consultation team (1 per week)	Support therapist’s motivation, adherence, and effectiveness	1) Mindfulness exercise 2) Clinical concerns, including therapist’s TIB

Mehlum and colleagues (see Table 4) ^120, 121^ completed a multisite RCT in Oslo, Norway, comparing 19 weeks of outpatient DBT with enhanced usual care (EUC; i.e. any non-DBT therapy plus suicide risk assessment). DBT was superior to EUC in reducing the frequency of SH behaviors, severity of SI, and depressive symptoms, with this DBT condition showing large effect sizes with regard to treatment outcome, whereas the EUC condition resulted in weak to moderate outcomes. At 1-year follow-up, DBT remained superior to EUC in reduction of SH frequency; however, there were no significant differences between treatment conditions for SI, hopelessness, depressive symptoms, and BPD features ^120, 121^.

**Table 4.** Mehlum *et al*. 2014 ^120^; 2016 ^121^.

Sample size (at randomization)	Sample characteristics	Recruitment setting	Inclusion criteria	Exclusion criteria	Major diagnoses
Total = 77; T = 39, C = 38	12–18 years old; 88% female; 85% Norwegian	Outpatient	Lifetime SH ≥2 episodes; ≥1 SH episode in past 16 weeks; ≥2 DSM-IV BPD criteria or 1 BPD criteria and 2 subthreshold-level criteria; fluent in Norwegian	BP; SZ; SCAD; psychotic disorder not otherwise specified; intellectual disability; Asperger syndrome	ANX (43%); other depressive disorder (38%); MDD (22%); BPD (21%); PTSD (17%); PD (9%); ED (8%); SUD (8%)
SH outcome measures	Treatment condition	Control condition	Assessments	Treatment attrition and completion	Results
SH (LPC); SI (SIQ-Jr)	DBT: individual sessions, multifamily skills training, family therapy, or telephone coaching as needed Dose: 19 weeks of weekly individual (1 hour) and multifamily skills training (1.5 hour)	EUC (any enhanced, non-DBT treatment plus suicide risk assessment, and therapist agrees to minimum dose) Dose: 19 weeks minimum of weekly individual sessions	Pretreatment (baseline), mid- treatment (9 and 15 weeks), post-treatment (19 weeks), and follow-up at 71 weeks (1 year post- treatment)	Treatment completion (≥50% of sessions): DBT: 74.4% EUC: 71% Attrition post-treatment: DBT: 0% EUC: 0% 71-week follow-up: DBT: 2.6% EUC: 2.6%	Significantly fewer SH episodes, significantly greater decrease in SI, significant decrease in depressive symptoms for DBT (compared to EUC) at post-treatment. Significantly fewer SH episodes from post- treatment to 71-week follow-up. NS between-group differences in SI, hopelessness, depressive symptoms, and BPD at 71-week follow-up.

ANX, anxiety disorder (type not specified); BP, bipolar disorder; BPD, borderline personality disorder; C, control condition; DBT, dialectical behavior therapy; DSM-IV, Diagnostic and Statistical Manual of Mental Disorders, 4 ^th^ edition; ED, eating disorder; EUC, enhanced usual care; LPC, lifetime parasuicide count; MDD, major depressive disorder; NS, non-significant; PD, personality disorder; PTSD, post-traumatic stress disorder; SCAD, schizoaffective disorder; SH, self-harm; SIQ-Jr, suicide ideation questionnaire, junior; SUD, substance use disorder; SZ, schizophrenia; T, treatment condition.

In the U.S., McCauley and colleagues (see Table 5) ^122^ conducted a multisite RCT with adolescents at high risk for suicide (i.e., with a history of at least one lifetime SA and repetitive SH) to determine whether DBT is effective in reducing SAs, in addition to SH. Participants received either 6 months of comprehensive DBT or individual and group supportive psychotherapy (IGST), a manualized, client-centered approach that included individual and group components. Internal validity was enhanced by IGST’s match to DBT for hours of treatment, modalities (i.e. both individual and group therapy), therapy drop-out policies, therapist expertise, and availability of supervision ^123^. Results demonstrated that youth in the DBT condition reported significantly fewer SAs, NSSI, total SH and SI at the end of the treatment than youth in IGST. While both groups continued to demonstrate improvements at 12-month follow-up, there were no significant differences between DBT and IGST ^122^. Taken together, these studies support DBT as the only replicated treatment with demonstrated efficacy at reducing SH in adolescents.

**Table 5.** McCauley *et al*. ^122^.

Sample size (at randomization)	Sample characteristics	Recruitment setting	Inclusion criteria	Exclusion criteria	Major diagnoses
Total=173; T=86, C=87	12–18 years old; 95% female; 56% Caucasian; 27% Hispanic; 7% African American; 6% Asian American; <1% Native American; 2% other	Emergency department, inpatient, outpatient, community	Lifetime SA ≥1; elevated SI over past month (SIQ-Jr ≥24); lifetime SH ≥3, with ≥1 SH episode in past 12 weeks; ≥3 BPD criteria	IQ <70; psychosis; mania; anorexia nervosa; life-threatening conditions; youth not fluent in English; parent not fluent in Spanish or English	MDD (84%); ANX (54%); BPD (53%); ED (<1%)
SH outcome measures	Treatment condition	Control condition	Assessments	Treatment attrition and completion	Results
SI (SIQ-Jr); SA (SASII); NSSI (SASII); SH (SASII)	DBT: individual sessions; multifamily skills training; youth and parent phone coaching; individual parent sessions; family sessions as needed Dose: 6 months of weekly individual and group sessions; weekly therapist team consultation	IGST: individual and group sessions, parent sessions (as needed) Dose: 6 months of weekly individual and group sessions; weekly therapist team consultation	Pretreatment (baseline), mid- treatment (3 months), post-treatment (6 months), follow-up at 9 and 12 months	Treatment completion (≥24 adolescent sessions): DBT: 45.4% IGST: 16.1% Attrition post-treatment: DBT: 10.5% IGST: 24.1% 12-month follow-up: DBT: 19.8% IGST: 26.4%	Significantly greater decrease in SI, SA, NSSI, and SH frequency for DBT (compared to IGST) at post- treatment NS between-group differences in SI, SA, NSSI, and SH from post- treatment to 12- month follow-up

ANX, anxiety disorder (type not specified); BPD, borderline personality disorder; C, control condition; DBT, dialectical behavior therapy; ED, eating disorder; IGST, individual and group supportive therapy; MDD, major depressive disorder; NS, non-significant; SA, suicide attempt; SASII, suicide attempt self-injury inventory; SH, self-harm; SI, suicide ideation; SIQ-Jr, suicide ideation questionnaire, junior; T, treatment condition.

In the McCauley *et al*. (2018) study, DBT was not superior to control at 12-month follow-up at reducing SA and SH ^122^, suggesting that more work is needed to determine how to sustain significant effects of DBT after active treatment has ended. Additionally, DBT is a time- and labor-intensive treatment. It generally requires a 4–6-month commitment from adolescents and families and includes at least two sessions per week (one individual session and one multifamily skills group). Further, DBT is a complex treatment that requires significant provider training. Thus, dismantling studies will be useful in determining what aspects of DBT yield significant effects, and establishment of shorter, less training-intensive treatments for SH/suicidal adolescents will be particularly useful for communities where—based on access to training or financial resources—comprehensive DBT training is not an option, and for those families who are unable to make the time and/or potential financial commitment DBT requires.

### Hospitalization

The AACAP’s *Practice Parameter for the Assessment and Treatment of Children and Adolescents with Suicidal Behavior* recommends that psychiatric hospitalization be considered if an adolescent is determined to be at high risk of imminent suicidal behavior ^45^. At present, however, there is no research supporting the effectiveness of hospitalization at reducing future SAs ^18, 117^. In fact, Chung and colleagues’ recent meta-analysis reviewed 100 studies of patients hospitalized for suicidality and found the highest rates of death by suicide in the first 3 months after discharge. These rates are 100 times the global suicide rate ^18^. Data have also shown that rehospitalization rates are high: 13% at 90-day follow-up ^124^, 38% at 6-month follow-up ^125^, and 30–43% at 1-year follow-up ^126^. These data suggest that hospitalization is not an adequate intervention for lowering risk for subsequent suicidal behavior ^29^ and, in fact, may confer greater risk for future suicidal behavior.

## Conclusion

Suicide is the second leading cause of death among adolescents ^3^. Not only is NSSI a risk factor for SAs in teens ^7, 8^ but also recent research revealed a temporal relationship between these behaviors, such that NSSI increases the likelihood of future SA, and SI often precedes the first onset of NSSI ^13^. While, by definition, the intent of NSSI is not death, these findings underline the importance of taking NSSI seriously and performing ongoing assessment of, and intervention for, suicidal behavior.

There are several evidence-based and evidence-informed interventions for managing suicide risk in adolescents (e.g. written safety plan, means restriction, and increased monitoring by a parent or trusted adult). While hospitalization is at times unavoidable for individuals who are unable to commit to the use of a written safety plan and have active SI with intent and plan, it has been found that risk for suicide increases significantly immediately post-hospitalization. Further work on how to enhance the effectiveness of hospitalization and to reduce the increased risk associated with it is a critical next step.

Overall, there is a small number of empirically supported treatments for decreasing SAs and NSSI in adolescents. DBT is the first and only well-established treatment (i.e. significant results compared to control in at least two RCTs performed by two independent research groups) ^116^ for SH adolescents at high risk for suicide ^127^. Replication trials are needed for several other treatments that have shown promise in decreasing SH in adolescents. Common elements of the interventions with significant results from at least one RCT included family involvement or support, emotion regulation skills, communication skills, and problem-solving skills; however, replications and dismantling studies are necessary to better understand the effects of these interventions and their components on reducing adolescent SH and SAs ^28^.
